# Mobility Control of Unmanned Aerial Vehicle as Communication Relay to Optimize Ground-to-Air Uplinks

**DOI:** 10.3390/s20082332

**Published:** 2020-04-19

**Authors:** Gaofeng Wu, Xiaoguang Gao, Kaifang Wan

**Affiliations:** School of Electronics and Information, Northwestern Polytechnical University, Xi’an 710129, China; wugaof@mail.nwpu.edu.cn (G.W.); wankaifang@nwpu.edu.cn (K.W.)

**Keywords:** unmanned aerial vehicle, relay, motion control, gradient methods, wireless communication

## Abstract

In recent years, unmanned aerial vehicles (UAVs) have been considered an ideal relay platform for enhancing the communication between ground agents, because they fly at high altitudes and are easy to deploy with strong adaptabilities. Their maneuvering allows them to adjust their location to optimize the performance of links, which brings out the relay UAV autonomous mobility control problem. This work addressed the problem in a novel scene with mobile agents and completely unknown wireless channel properties, using only online measured information of received signal strength (RSS) and agent positions. The problem is challenging because of the unknown and dynamic radio frequency (RF) environment cause by agents and UAV maneuvering. We present a framework for both end-to-end communication and multi-agent-inter communication applications, and focus on proposing: (1) least square estimation-based channel approximation with consideration of environment effects and, (2) gradient-based optimal relay position seeking. Simulation results show that considering the environmental effects on channel parameters is meaningful and beneficial in using UAV as relays for the communication of multiple ground agents, and validate that the proposed algorithms optimizes the network performance by controlling the heading of the UAV.

## 1. Introduction

### 1.1. Background

In recent decades, using coordinated multiple agents to realize an objective has shown great superiority in various tasks, which are extremely varied and include missions such as civilian search and rescue [1], environment sensing and monitoring [2], surveillance and reconnaissance [3,4], wildland firefighting [5], and other scientific research. In such Multi-Agent Systems (MAS), mission efficiency is severely affected by the quality of information exchange. Such applications often occur on demand and in environments without fixed communication infrastructures, and thus the network must be wireless, and operate in a peer-to-peer or ad hoc manner [6]. However, increasing distance and obstructing due to terrain or surroundings (such as trees, building, etc.) negatively affects the quality of wireless communication severely because signal strength decreases exponentially [7,8].

Addressing these, communication relays have been deployed to support information exchange [9]. Compared to satellite platforms and ground vehicles [10,11], unmanned aerial vehicles (UAVs) can fly at high altitude so as to provide Line of Sight (LoS) or near Line of Sight communication [6] with higher possibility. This makes using UAVs as communication relay much more appropriate and ideal. More importantly, such on-the-fly and unmanned platforms also show their high adaptability and survivability in harsh environments, giving them the possibility of being generally and widely applied.

One typical application is using UAV as communication relay flying over a collection of multiple ground agents, such as wireless sensors and nodes in internet of things, receiving messages from these ground agents and forward them to other agents. Normally, the agent’s transmit power is limited and low, much smaller than the relay transmit power, and signal power decreases exponentially and may be affected by environment obstacles, the ground-to-air uplink channels qualities from agent to relay UAV should be optimized. A novel technology is taking advantage of the UAVs’ maneuvering ability, namely optimizing the ground-to-air uplink channels by controlling the mobility of relay UAV. However, the mobility control problem is co-related to wireless signal propagation properties, which is influenced by environmental effects, making the controlling the mobility of relay UAV a real challenge. Next, we review some of works related to the relay UAV mobility control problem.

### 1.2. Related Work

There is increasing interest in using UAVs as communication relays and a variety of approaches was proposed to optimize the performance of the UANet. Yuan et al. [12] proposed a motion-planning method to optimize end-to-end bit error rate (BER) with using a stochastic channel approximation algorithm [13]. Ono et al. [14] focused on designing the turning radius and maneuvering altitude of the UAV for realize communication between multiple ground nodes in the event of disasters by proposing a variable-rate relaying approach. Mozaffari et al. [11] studied achieving energy-efficient data collection in Internet of Things (IoT) applications by proposing a framework to deploy the UAVs at appropriate locations and control their mobilities. Wang and Ren [15] presented a joint transmit power and trajectory optimization algorithm for the relay UAV-based non-convex optimization. Krijestorac and Hanna [16] studied the issue of placing UAV as relay to connect to a user with unknown position using deep reinforcement learning (deep RL) method. Sharma and Kim [17] proposed a mixed mobility (MM) model which characterizes the movement process of a UAV in the 3D cylindrical region by invoking the random waypoint mobility (RWPM) and uniform mobility (UM) models to represent the movement of a UAV in vertical and spatial directions. However, users in these works are required to be static, and this cannot be satisfied in many UAV assisted MAS applications because the motion of agents is often decided by missions.

The others focused on using UAVs as relays for mobile users. Their approaches can be summarized and grouped into two categories: considering only path loss [18,19,20,21,22], and considering fading effects. Channel models in the former works are over-simplified, and would result in inferior network performance. Thus, we focus on discussing research that consider channel fading effects when controlling relay UAV mobility for mobile users.

A correlated Rician fading channel model between each ground node and the UAV was studied by Jiang and Swindlehurst [6], while a Rayleigh case was studied by Zhan and Yu [23,24], which could be summarized as Probabilistic Channel Models (PCMs). Although our previous work [25] studied controlling the mobility of relay UAV for multiple mobile users, they are airborne users and channel fading are main composed of large scale fading, while in realistic ground-to-air wireless communication, the signals could be shadowed or reflected by obstructs, such as terrains, buildings, trees. Thus, none of the above-mentioned works considered the effect of the environment (such as building density in urban environment) on wireless channels, especially the occurrence of LoS components [26].

Some other researchers, such as in References [26,27], considered environmental effects on the ground-to-air uplink properties; they assumed these channel parameters are available and identical. Michailidis et al. [28] proposed a three-dimensional (3-D) geometry-based optimization method for the relay location in an airborne multi-user multiple-input multiple-output (MIMO) communication system. The proposed method generated required control command, including azimuth angle, and elevation angle, by giving out their analytic expression, which is similar to [22]. Channel parameters in both works are assumed known and identical, such as the path-loss exponent is set as 2. Ladosz and Oh [29] proposed to integrate a learning-based measurement technique to predict channels; they still assumed the channel parameters for different environment types are previously known. Actually, these are unrealistic because in on-demand application regions, these parameters are often location dependent, and changing with the maneuvering of agents.

Dixon and Frew [30,31,32] proposed a decentralized data-driven chain controller by driving the relay UAV to loiter at specific positions for obtaining perturbed objective for distanced agents, This method could not be applied to multiple user situations, and requires quasi-static RF distribution. It is unsatisfied in realistic applications where the mobilities of users is determined by correlated missions.

To the best of the authors’ knowledge, no previous work has ever studied autonomous control of the mobility of relay UAV for serving mobile ground agents with previously unknown and dynamic radio frequency environment.

### 1.3. Contribution

Motivated by the aforementioned observations, this paper investigates using a UAV as communication relay serving multiple ground agents communication. This scenario may refer to areas such as wireless sensor networks (WSN) and IoT. In particular, an average-gain channel model (ACM) is considered to represent the ground-to-air uplinks, where the environment affects on LoS probability is reflected. The main challenges for the UAV autonomous mobility control are mobile agents and prior unknown and dynamic RF distributions. A novel mobility control framework is proposed, where the UAV flies at a fixed altitude with constant speed, leaving the control command as its turn rate. The framework decouples the problem by generating the optimal relay position first, and engaging a guidance law so as to continuous give out required turn rate. To address the former problem, this paper focuses on the following two main contributions: (1) A least-square estimation-based channel approximation method is proposed, where only the latest online sensed RSS and position information. Hereby, the positions of agents are estimated and predicted using a Kalman filter; (2) We study the mobility control method in both relay for end-to-end communication and multi-agent-inter communication scenarios, where the former contains only two agents and the latter contains multiple agents, respectively. Gradient-based methods instead of global search of the optimal relay position are proposed. The proposed mobility control progress is real-time and autonomous.

### 1.4. Structure

This work addresses the problem in a novel scene with mobile agents and completely unknown wireless channel properties, only using online measured information of received signal strength (RSS) and agent positions. We organize this paper as follows: Section 2 formulates the problem and briefly describes the method framework and our contribution. Section 3 further presents the KF-based algorithm for prediction the positions for the agents, and the LVGF-based guidance law for generating desired turn rate. Section 4 first presents the online channel approximation method, then presents the methods for seeking optimal relay position with unkownn channel parameters for the UAV. Section 5 tests the proposed methods via multiple simulation with analyzed results. Section 6 summarizes this paper with conclusions.

## 2. Problem Formulation and Method Framework

Consider *N* mobile and networked ground agents $u_{i}\in U=\left\{u_{1},u_{2},\ldots ,u_{N}\right\}$ with position $P=\{p_{1},p_{2},\ldots ,p_{N}\}$ carrying out tasks in region $D\subset R^{2}$, each agent is equipped with communication devices with limited and low performance. To address the negative impact (as discussed in Section 1) of distance and obstacles to the quality of wireless channel, one relay UAV equipped with higher performance communication relay devices are expected to be deployed and controlled to provide optimized network performance.

### 2.1. Relay Uav Mobility Control Problem

In this work, the ground users carry out tasks, such as search and rescue, in the region. Their trajectories are decided by their decision makers, unknown and cannot be affected by the UAV. Assuming the UAV operates at constant height and speed (which is reasonable for many cases), and the time for UAV to achieve required turn rate and air speed is fast enough to be neglected because of its fast-inner loop autopilot system. Thus, a standard (Cartesian) bicycle-like model [33,34] as follows can be used to denote the UAV kinematic:

(1) $$\begin{array}{cc} \; & \dot{x}=v\cos\psi \\ \; & \dot{y}=v\sin\psi \\ \; & \dot{\psi }=\omega \;\;\left|\omega \right|\leq \omega_{\max} \end{array}.$$

where $p={\left[x,y\right]}^{T}\in D$ is the position vector of the UAV, $v={\left[\dot{x},\dot{y}\right]}^{T}$ is the speed vector, $\psi \in \left[0,2\pi \right)$ is the heading angle, v=|v| is constant as aforementioned. Owing to vehicle operational performance constraints, turn rate of the UAV is limited [30]:

(2) $$\omega \leq \frac{g\tan\left(\phi_{\max}\right)}{v}.$$

where ϕ is the bank angle, *g* is the gravitational acceleration.

As this work attempts to optimize the performance of the relay network through controlling the motion of the UAV, and mobility of the UAV could only be optimized through controlling its turn rate $\dot{\psi }$, the mobility control optimization problem can thus be given as:

(3) $${\dot{\psi }}^{*}=\arg\max_{|\dot{\psi }|\leq \omega_{\max}}J\left(\dot{\psi }\right)$$

where $J(\dot{\psi })$ represents the network performance, reflecting the quality of ground-to-air uplinks.

The RF distribution of the environment is directly related to the position of the UAV, while the controlling variable is its turn rate, this makes Equation (3) difficult to solve. To decouple the objective function *J* from $\dot{\psi }$, this work attempts to seek the optimal relay position for the UAV first, and then use a guidance law to achieve desired $\dot{\psi }$ so as to drive the relay UAV this position. Thus, the problem in Equation (3) could be reformulated as:

(4) $$p^{*}=\arg\max_{p\in D}J\left(p\right)$$

where $p^{*}$ represents the optimal relay position.

This work considers two communication scenarios of using UAV as relays for multiple ground agent: end-to-end, and multi-agent inter communication.

**Figure 1:**
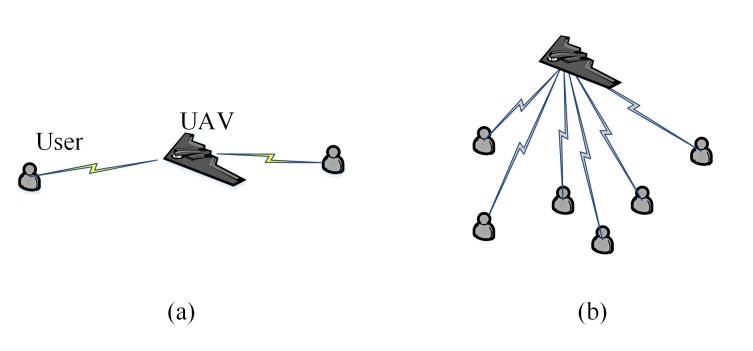
Network structures: (**a**) end-to-end communication; (**b**) multi-agent inter communication.

In end-to-end communication [35], as shown in Figure 1a, there includes two disconnected agents. A representative application is that two agent execute mission in far-field region, sharing information and decisions with each other so as to improve mission efficiency. The relay UAV is deployed to improve their communication quality. Because the communication ability of the UAV is much better than the agents, communication quality between either agent and the UAV is normally constrained by the channel from the agent to the UAV, denoted as uplink channel. Then Equation (4) can be reformulated as follows because their communication quality is limited to the worse uplink:

(5) $$p^{*}=\arg\max_{p\in D}\min\left\{S_{p_{1},p},S_{p_{2},p}\right\},\;p_{1},p_{2}\in D$$

where $J=\min\{S_{p_{1},p},S_{p_{2},p}\}$ is the network performance and objective metric in relay for end-to-end communication, and the higher value of *J* means the better communication quality between the two agents.

In multi-agent inter communication, as shown in Figure 1b, any couple of agents in the network may requires to exchange message, using the min(·) function as (5) as objective is unsuited. However, not all the uplinks could be optimized synchronously, and it may be impossible for the UAV to promote the qualities of all links to the level satisfying message exchange, this work uses the follow function as objective to globally reflects network performance by referring to References [22,36]:

(6) $$p^{*}=\arg\min_{p\in D}\sum_{i=1}^{N}\frac{1}{S_{p_{i},p}}$$

where $J=\sum_{i=1}^{N}\frac{1}{S_{p_{i},p}}$ is the network performance and objective metric in relay for multi-agent-inter communication, and the lower value of *J* means the better communication quality among these agents in the mass.

### 2.2. Mobility Control Framework

The primary goal of this work is the mobility control methods to drive the relay UAV to appropriate position for optimized objective function, by jointly considering the unknown multi-agent mobilities, the environment effects on the channel properties, and the completely prior unknown channel parameters. Since the angle of arrival (AoA) signal [22] is hard to obtain with unidirectional antenna, the only available data for the mobility control process in the received signal strength (RSS) at the receiver antenna of the UAV, and agent positions, which can be obtained easily through the global positioning system (GPS).

**Figure 2:**
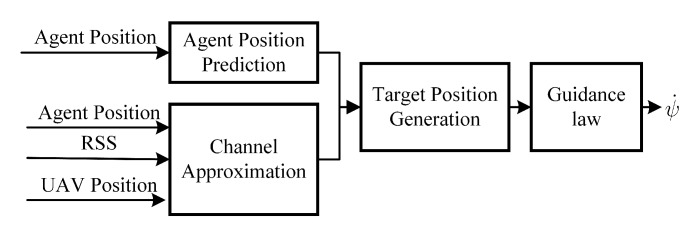
Mobility control framework.

To address the mobility control problem of the UAV, this work proposes a mobility control framework as shown in Figure 2, which main contains four parts: agent position prediction, online channel approximation, optimal relay position generation, and the guidance law. The main contributions and concentrations of this work are: (1) online channel approximation: a channel approximation method based on least square estimation (LSE) algorithm using latest online sensed RSS to address the problem of completely prior unknown channel parameters. (2) optimal relay position generation: gradient-based methods instead of global search of the optimal relay position is proposed to address the problem of real-time autonomous optimization, while the other two parts of agent position prediction and guidance law are solved using existing methods, namely the Kalman-filter method and Lyapunov Guidance Vector Field based controller.

## 3. Position Prediction and Guidance Law

From Equation (4), it is easy to find that: (1) The optimal relay position is correlated with the position of agents, however, the informed agent positions are normally inaccurate, and provide no future position information. (2) The output by solving Equation (4) is not the required turn rate of the UAV as aforementioned. In this section, a Kalman-Filter (KF) [37] based position prediction method and a Lyapunov Guidance Vector Field (LGVF) [38,39] controller will be consecutively discussed to address the two above problems respectively.

### 3.1. Kf-Based Position Prediction

Assume that the motion of agents could be simulated using a first order auto-regressive (AR) model, and let $s_{i,k}=\left[x_{i,k},y_{i,k},{\dot{x}}_{i,k},{\dot{y}}_{i,k}\right]$ denotes the state of agent $u_{i}$ at time instance $t_{k}$, where ${\dot{x}}_{i,k}$ and ${\dot{y}}_{i,k}$ denote the velocities of agent $u_{i}$, then the standard KF could be applied as follows:

The state transition equation from time $t_{k}$ to $t_{k+1}$ is:

(7) $$s_{i,k}=Fs_{i,k-1}+\xi_{i,k-1},\;F=\left[\begin{array}{cccc} 1 & 0 & \Delta t & 0\\ 0 & 1 & 0 & \Delta t\\ 0 & 0 & 1 & 0\\ 0 & 0 & 0 & 1 \end{array}\right]$$

where F is the state transition matrix, $\Delta t=t_{k}-t_{k-1}$, $\xi_{i,k-1}$ is the process noise.

The observation equation at time $t_{k}$ is:

(8) $$z_{i,k}=Ts_{i,k}+\nu_{i,k},\;T=\left[\begin{array}{cccc} 1 & 0 & 0 & 0\\ 0 & 1 & 0 & 0 \end{array}\right]$$

where T is the observation matrix, and $\nu_{i,k}$ is the observation noise.

Then the KF procedures can be given as follows:

Initialization:

(9) $${\hat{s}}_{i,0}=E\left[s_{i,0}\right],\;P_{i,0}=\left[\begin{array}{cccc} 0 & 0 & 0 & 0\\ 0 & 0 & 0 & 0\\ 0 & 0 & 0 & 0\\ 0 & 0 & 0 & 0 \end{array}\right]$$

Prediction:

(10) $${\hat{s}}_{i,k|k-1}=F{\hat{s}}_{i,k-1}$$

(11) $$P_{i,k|k-1}=FP_{i,k-1}F^{T}+Q_{i,k-1}$$

Kalman gain:

(12) $$K_{i,k}=P_{i,k|k-1}T^{T}{\left(TP_{i,k|k-1}T^{T}+R_{i,k}\right)}^{-1}$$

State measurement and covariance matrix:

(13) $${\hat{s}}_{i,k}={\hat{s}}_{i,k|k-1}+K_{i,k}\left(z_{i,k}-T{\hat{s}}_{i,k|k-1}\right)$$

(14) $$P_{i,k}=\left(I_{4}-K_{i,k}T\right)P_{i,k|k-1}$$

### 3.2. Lgvf Guidance Law

The LGVF controller as presented by Frew et al. [38,39] is used in this work to generate desire turn rate $\dot{\psi }$ so as to drive the UAV to the optimal relay position $p^{*}={\left[x^{*},y^{*}\right]}^{T}$. Let $r=p-p^{*}={\left[x^{r},y^{r}\right]}^{T}$, representing the relative position of UAV’s current position to $p^{*}$. Then rewrite its kinematic model as follows:

(15) $$\begin{array}{c} {\dot{x}}^{r}=v\cos\psi -{\dot{x}}^{*}=v^{r}\cos\eta \\ {\dot{y}}^{r}=v\sin\psi -{\dot{y}}^{*}=v^{r}\sin\eta \\ \dot{\psi }=\omega =\dot{\eta }/k_{\eta }\left(\psi \right) \end{array}$$

where $v^{r}=∥\dot{r}∥$, η, $k_{\varepsilon }\left(\psi \right)$ can be calculated as follows:

(16) $$v^{r}{}^{2}=v^{2}+{\dot{x}}^{*}{}^{2}+{\dot{x}}^{*}{}^{2}-2v\left({\dot{x}}^{*}\cos\psi +{\dot{x}}^{*}\sin\psi \right)$$

(17) $$\varepsilon =\arctan\frac{{\dot{y}}^{r}}{{\dot{x}}^{r}}$$

(18) $$k_{\varepsilon }\left(\psi \right)=\frac{v^{2}-v\left({\dot{x}}^{*}{}^{2}\cos\psi +{\dot{y}}^{*}{}^{2}\sin\psi \right)}{v^{r}{}^{2}}$$

Minimizing the Lyapunov function $l\left(\dot{r}\right)={\left(∥\dot{r}{∥}^{2}-r^{d}{}^{2}\right)}^{2}$, provides the desired turn rate [30]:

(19) $$\dot{\psi }=-K_{l}\langle \eta -\eta^{d}\rangle +\frac{{\dot{\eta }}^{d}}{k_{\eta }\left(\psi \right)}$$

where $r^{d}$ is the desired loitering radius at $p^{*}$, and:

(20) $$\begin{array}{cc} \eta^{d}\; & =\arctan\left(\frac{y\cdot \left(r^{2}-r^{d}{}^{2}\right)-x\cdot 2rr^{d}}{x\cdot \left(r^{2}-r^{d}{}^{2}\right)+y\cdot 2rr^{d}}\right) \end{array}$$

With using the kinematic constraint $-\pi <\langle \eta -\eta^{d}\rangle \leq \pi$, gives control command:

(21) $$\left|{\dot{\psi }}^{c}|=\max(\omega_{\max},\left|\dot{\psi }\right|\right)$$

## 4. Optimal Relay Position Generation with Unknown Channel Parameters

In the majority of applications, parameters of wireless channels are hardly known to the decision makers, because of the dynamic, complicated environment, in which scenarios, estimation of channel parameters should be firstly executed before further discussion of the mobility control problem.

### 4.1. Channel Approximation

As discussed in Section 1, none of the Probabilistic Channel Model (PCM) synthetically considering the path loss, shadowing and multi-path effect has shown its capability in describing the effect of the environment on channel properties [26]. This work studies the network optimization problem in urban environment, and applies an Average-gain Channel Model (ACM) where building distributions and properties are considered.

The groups of received ground-to-air signal (LoS, NLoS, multipath fading) can be considered separately with different probabilities of occurrence as shown in References [40,41]. Typically, it is assumed that the received signal is categorized in only one of those groups [42]. Each group has a specific probability of occurrence which is a function of environment, density and height of buildings, and elevation angle. Please note that the probability of having the multipath fading is significantly lower than the LoS and NLoS groups [42]. Therefore, the impact of small-scale fading can be neglected in this case [40]. One common approach for modeling air-to-ground propagation channel is to consider LoS and NLoS components along with their occurrence probabilities separately as follows [26,40,41,42]:

(22) $$L_{i}=\left\{\begin{array}{cc} \eta_{1}{\left(\frac{4\pi f_{c}d_{i}}{c}\right)}^{\kappa }, & \mathrm{LoS}\;\mathrm{link},\\ \eta_{2}{\left(\frac{4\pi f_{c}d_{i}}{c}\right)}^{\kappa }, & \mathrm{NLoS}\;\mathrm{link} \end{array}\right.$$

where $L_{i}$ is the average path loss between the relay UAV and $u_{i}$, $f_{c}$ is the carrier frequency, κ is the path loss exponent varies 2–6 [8], $\eta_{1}$ and $\eta_{2}$ ($\eta_{2}>\eta_{1}$) are the excessive path loss coefficients in LoS and NLoS cases, *c* is the speed of light, and $d_{i}$ is the distance between $u_{i}$ and the UAV.

Typically, given only the locations of the UAVs and devices, it is not possible to exactly determine which path loss type (LoS/NLoS) is experienced by the device-UAV link. In this case, the path loss average considering both LoS and NLoS links can be used for the device-UAV communications. The average path loss between $u_{i}$ and *r* can be expressed as Reference [11]:

(23) $$\begin{array}{cc} {\bar{L}}_{i}\; & =p_{i}^{\mathrm{LoS}}\eta_{1}{\left(\frac{4\pi f_{c}d_{i}}{c}\right)}^{\kappa }+p_{i}^{\mathrm{NLoS}}\eta_{2}{\left(\frac{4\pi f_{c}d_{i}}{c}\right)}^{\kappa }\\ \; & =\left[p_{i}^{\mathrm{LoS}}\eta_{1}+p_{i}^{\mathrm{NLoS}}\eta_{2}\right]{\left(K_{0}d_{i}\right)}^{\kappa } \end{array}$$

where $K_{0}=\frac{4\pi f_{c}}{c}$, $p_{i}^{\mathrm{LoS}}$ is the LoS probability, and $p_{i}^{\mathrm{NLoS}}$ is the NLoS probability with expression:

(24) $$p_{i}^{\mathrm{NLoS}}=1-p_{i}^{\mathrm{LoS}}$$

**Figure 3:**
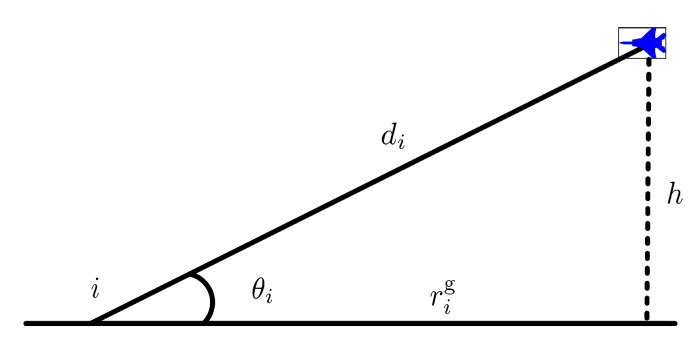
Parameters in ground-to-air channel.

The effect of the environment on the probability of LoS is remarkably found as a function of the transmitter elevation $h^{\mathrm{TX}}$ and receiver elevation $h^{\mathrm{RX}}$, and depends on the environmental statistical parameters, according to the International Telecommunication Union (ITU) in its recommendation document [7], and can be write as [42]:

(25) $$p^{\mathrm{LoS}}=\prod_{k=0}^{m}\left[1-\exp\left(-\frac{{\left[h^{\mathrm{TX}}-\frac{\left(n+\frac{1}{2}\right)\left(h^{\mathrm{TX}}-h^{\mathrm{RX}}\right)}{m+1}\right]}^{2}}{2\gamma^{2}}\right)\right]$$

where $m=\mathrm{floor}(r^{g}\sqrt{\alpha \beta }-1)$, α is the ratio of built-up land area to the total land area (dimensionless), β is the mean number of buildings per-unit area (buildings/km${}^{2}$), γ is a scale parameter that describes the buildings height distribution according to Rayleigh probability density function: $f\left(H\right)=\left(H/\gamma^{2}\right)\exp\left(-H^{2}/2\gamma^{2}\right)$, where *H* is the building height in meters, $r^{g}$ is the ground distance between the transmitter and the receiver, as depicted in Figure 3.

Equation (25) is independent of the system frequency, and generically can be used for any $h^{\mathrm{TX}}$ and $h^{\mathrm{RX}}$. Since $h^{\mathrm{TX}}$ is much lower than the average building heights and UAV latitude, then the ground distance becomes $r^{g}=h/\tan\left(\theta \right)$ by disregarding $h^{\mathrm{TX}}$, where *h* is the UAV altitude. Reference [42] showed that Equation (25) can be closely approximated to a simple modified Sigmoid function (S-curve) of the following form:

(26) $$p^{\mathrm{LoS}}=\frac{1}{1+C\exp(-B(\theta -C))}$$

where *B* and *C* are called here the S-curve parameters, and:

(27) $$\theta_{i}=\frac{180}{\pi }\times \arcsin\left(\frac{h_{i}}{d_{i}}\right)$$

Clearly, the average channel gain between the UAV and the device is:

(28) $${\bar{g}}_{i}=\frac{1}{{\bar{L}}_{i}}$$

The qualities of wireless communication channels, such as capacity, delay, bit-error-rate, etc. are closely related to RSS [8], they are used as the channel performance metric in this work and could be given as follows:

(29) $$S\left(p_{i},p\right)=P_{i}^{T}\cdot {\bar{g}}_{i}$$

where $S\left(p_{i},p\right)$ represents the RSS of *r* at position p from agent $u_{i}$ at position $p_{i}$, $P_{i}^{T}$ is the transmitter power of agent $u_{i}$.

Ground-to-air uplink channel properties are correlated with LoS probabilities, environmental properties, user-to-UAV elevation angle and distance. These make the channel estimation progress difficult. This work starts solving the problem from reformulating the average path loss model in Equation (23) as follows:

(30) $$\begin{array}{cc} P_{i}^{r} & =\frac{P_{i}^{T}}{\left(\eta_{1}p_{i}^{\mathrm{LoS}}+\eta_{2}p_{i}^{\mathrm{NLoS}}\right){\left(K_{0}d_{i}\right)}^{\kappa }}\\ \; & =\frac{P_{i}^{T}\left(1+C_{i}e^{B_{i}C_{i}-B_{i}\theta_{i}}\right)}{\left(\eta_{1}+\eta_{2}C_{i}e^{B_{i}C_{i}-B_{i}\theta_{i}}\right)K_{0}^{\kappa }d_{i}^{\kappa }} \end{array}$$

This equation shows that the channel is correlated with *B*, *C*, $\eta_{1}$, and $\eta_{2}$, which decides the LoS possibility in the ground-to-air average gain channel. In realistic applications, estimation the channel parameters in Equation (30) is hard because of too many of them. Since the transmission power of the agents can be easily obtained, the estimation progress turns to estimate parameters in the following equation:

(31) $$\begin{array}{cc} q_{i}^{r} & =\left(\eta_{1}p_{i}^{\mathrm{LoS}}+\eta_{2}p_{i}^{\mathrm{NLoS}}\right){\left(K_{0}\right)}^{\kappa }\\ \; & =\frac{\eta_{1}+\eta_{2}C_{i}e^{B_{i}C_{i}-B_{i}\theta_{i}}}{1+C_{i}e^{B_{i}C_{i}-B_{i}\theta_{i}}}{\left(K_{0}\right)}^{\kappa } \end{array}$$

Then Equation (30) can be reformulated as $P_{i}^{r}=P_{i}^{T}/\left(q_{i}^{r}d_{i}^{\kappa }\right)$, and by taking logarithm to it, there exists:

(32) $$P_{i}^{r}{}_{\mathrm{dB}}=10\log_{10}\left(\frac{P_{i}^{T}}{q_{i}^{r}}\right)-\kappa d_{i}{}_{\mathrm{dB}}$$

Considering the fact that in a small scale sampling period, the transmitting power of the agents could be shown as constant, similarly as *B*, *C*, $\eta_{1}$ and $\eta_{2}$, which are only affected by the position of each agent. The only impact factor to Equation (31) is $\theta_{i}$, normally which changes little, and with rather limited affects to $q_{i}^{r}$. As a result, $P_{i}^{r}{}_{\mathrm{dB}}$ could be approximated as linear proportional to $d_{i}{}_{\mathrm{dB}}$ as follows:

(33) $${\hat{q}}_{i}{}_{\mathrm{dB}}^{r}=\beta_{1}+\beta_{2}d_{i}{}_{\mathrm{dB}}$$

Actually, because $0\leq p_{i}^{\mathrm{LoS}}\leq 1$ and $p_{i}^{\mathrm{NLoS}}=1-p_{i}^{\mathrm{LoS}}$, $K_{0}^{\kappa }\eta_{1}\leq q_{i}^{r}\leq K_{0}^{\kappa }\eta_{2}$, which is bounded between two functions and ensures the approximation error is bounded. Using the least square estimation (LSE) method, the coefficients $\beta_{1}$ and $\beta_{2}$ can be generated as follows:

(34) $${\left[\beta_{1},\beta_{2}\right]}^{T}={\left(X^{T}X\right)}^{-1}X^{T}Y$$

where $X={\left[1,10\log_{10}d_{1},\ldots ,10\log_{10}d_{k}\right]}^{T}$ is the *k* times sampling of the agent positions, $Y={\left[P_{1}^{r}{}_{\mathrm{dB}},\ldots ,P_{k}^{r}{}_{\mathrm{dB}}\right]}^{T}$ is the sampled RSS related to X.

**Figure 4:**
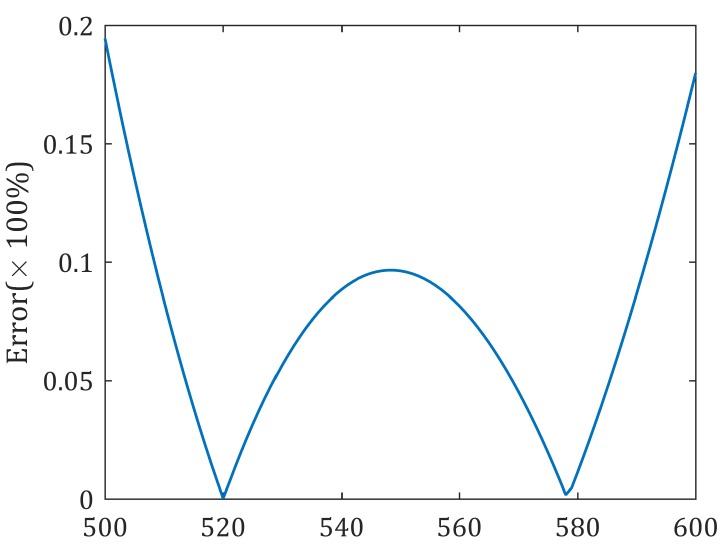
Approximation error of ground-to-air uplink channel in a small-scale district.

It should be noted out that $q_{i}^{r}$ in the aforementioned approximation is treated as constant in a small-scale district; this requires that environment type in a small sample period does not change. Fortunately and in most applications, the environment type only changes occasionally or not too often. In such scenarios, the relay UAV only use recently sampled data sets to estimate the channel model around present position. Figure 4 shows that the approximation error is less than 0.2% in an urban environment with parameter $\eta_{1}=1.6$, $\eta_{2}=23$, B=0.11, C=12.08, $f_{c}=2\mathrm{GHz}$, *h* = 400 m, and the distance varies from 500–600 m.

### 4.2. End-To-End Communication Scenario

In Section 4.1, an approximately estimation method of channel model in a small-scale district has been given, then the RSS model are rewrite as follows:

(35) $${\hat{S}}_{p_{1},p}{}_{\mathrm{dB}}={\hat{P}}_{i}^{r}{}_{\mathrm{dB}}=\beta_{2}d_{i}{}_{\mathrm{dB}}+\beta_{1}$$

which is continuous and differentiable.

Gradient direction is the fastest direction to improve objective function, so that network performance can be optimized with a gradient ascend-based algorithm. According to Equation (5), the use of min(·) function shows that *J* is not smooth, then derivation of objective function *J* needs to be discussed conditionally:

(36) $$\nabla J=\left\{\begin{array}{cc} \nabla S_{p_{1},p}, & \;\mathrm{if}\;S_{p_{1},p}<S_{p_{2},p}\;\\ \nabla S_{p_{2},p}, & \;\mathrm{if}\;S_{p_{1},p}>S_{p_{2},p}\;\\ \mathrm{Other}, & \;\mathrm{if}\;S_{p_{1},p}=S_{p_{2},p}\; \end{array}\right.$$

The gradient-based method does not use the gradient in Equation (36) directly, but instead follows with its direction using the following function:

(37) $$\mathrm{dir}\left(v\right)=v/{∥v∥}_{2},\;\mathrm{if}{∥v∥}_{2}\neq 0$$

and returns a target point in this direction instead of iteratively search the realistic optimal relay position, that is:

(38) $${\hat{p}}_{k+1}^{*}=p_{k}+G*\mathrm{dir}\left(v_{k}\right)$$

where *G* is the feedback gain, φ=dir(·) is the horizontal line-of-sight direction of $p^{*}$. The reason to use dir(·) function and Equation (38) are that: (1) the approximated channel does not suit for far-away fields, and (2) avoid to select appropriate *G* for every new environment.

However, the gradient direction is local optimal, the generated fly path may curved even in free space. To improve its performance, theorem 1 and its proposition are given first.

**Theorem** **1.** *If $r_{1}^{g}=0$ and $S_{p_{2},p}>S_{p_{1},p}$, then $p^{*}=\left(x_{1},y_{1},h\right)$; similar conclusion could be driven with $r_{2}^{g}=0$ and $S_{p_{1},p}>S_{p_{2},p}$.*

**Proof** **of** **Theorem** **1.** $r_{1}^{g}=0$ represents $d_{1}$ is minimized, and $\theta_{1}=\pi /2$, so that $p_{1}^{\mathrm{LoS}}\approx 1$. According to Equation (23) and Equation (26), $S_{p_{1},p}$ is maximized. Further with $S_{p_{2},p}>S_{p_{1},p}$ and Equation (36), the objective function is maximized optimal, which proves $p^{*}=\left(x_{1},y_{1},h\right)$, where $\left(x_{1},y_{1}\right)$ is the horizontal position of $u_{1}$. □

**Proposition** **1.** *If $r_{1}^{g}=0$, $S_{p_{2},p}\leq S_{p_{1},p}$ and $r_{2}^{g}=0$, $S_{p_{1},p}\leq S_{p_{2},p}$, seeking position $p^{*}$ equals to seek one position p satisfying: (1) $S_{p_{1},p}=S_{p_{2},p}$, and (2) $p=p_{1}+\lambda \left(p_{2}-p_{1}\right)$ (or $p=p_{2}+\left(1-\lambda \right)\left(p_{1}-p_{2}\right)$), where 0<λ<1.*

**Proof** **of** **Proposition** **1.** Situation (2) means the optimal relay position p locates in segment $\left[p_{1},p_{2}\right]$ defined by $p_{1}$ and $p_{2}$. The proposition is proved from the following two aspects:

(1) Proof of sufficiency: if $p=p^{*}$, then $S_{p_{1},p}=S_{p_{2},p}$ and $p=p_{1}+\lambda \left(p_{2}-p_{1}\right)$. Assuming $S_{p_{1},p}>S_{p_{2},p}$, then $\nabla S_{p_{2},p}=0$ because of Equation (39) and $\nabla J=\nabla S_{p_{2},p}$. $\nabla S_{p_{2},p}=0$ can be reached only when $r_{2}^{g}=0$, which means the UAV is directly above $u_{2}$ and RSS is maximized. It goes against the condition that if $r_{2}^{g}=0$, $S_{p_{1},p}\leq S_{p_{2},p}$, thus the assumption is not valid, meaning $S_{p_{1},p}\leq S_{p_{2},p}$. Similarly, $S_{p_{1},p}\geq S_{p_{2},p}$. Thus, $S_{p_{1},p}=S_{p_{2},p}$. Next, assume $p^{*}\notin \left[p_{1},p_{2}\right]$. It is definitely that there exits one position $p^{\prime }\in \left[p_{1},p_{2}\right]$ satisfying $S_{p^{\prime },p_{1}}=S_{p^{\prime },p_{2}}$, then $S_{p_{1},p^{*}}=S_{p_{2},p^{*}}\geq S_{p_{1},p^{\prime }}=S_{p_{2},p^{\prime }}$. This reaches $d_{p_{1},p^{*}}\leq d_{p_{1},p^{\prime }}$ and $d_{p_{2},p^{*}}\leq d_{p_{2},p^{\prime }}$ since parameters in Equation (23) are unrelated to the position of the UAV. This results in $d_{p_{1},p^{*}}+d_{p_{2},p^{*}}\leq d_{p_{1},p^{\prime }}+d_{p_{2},p^{\prime }}=d_{p_{1},p_{2}}$ according to the triangle properties. As the assumption not true, there must $p_{u}^{*}\in \left[p_{1},p_{2}\right]$.

(2) proof of necessity: if $S_{p_{1},p}=S_{p_{2},p}$ and $p\in \left[p_{1},p_{2}\right]$, then $p=p^{*}$. Supposing there exists another position $p^{\prime }$ satisfies $S_{p_{1},p^{\prime }}=S_{p_{2},p^{\prime }}$ and $p^{\prime }=p_{1}+\lambda \left(p_{2}-p_{1}\right)$, then $S_{p_{1},p^{*}}=S_{p_{2},p^{*}}>S_{p_{1},p^{\prime }}=S_{p_{2},p^{\prime }}$ as $p^{*}$ optimal. Thus, $d_{p_{1},p^{*}}<d_{p_{1},p^{\prime }}$, since channel parameters in Equation (23) are unrelated to the position of the UAV. Because $d_{p_{2},p_{1}}$ is constant, $d_{p_{2},p^{\prime }}=d_{p_{2},p_{1}}-d_{p_{1},p^{\prime }}<d_{p_{2},p^{*}}$, resulting in $S_{p_{2},p^{\prime }}>S_{p_{2},p^{*}}$. This contradict $S_{p_{2},p^{\prime }}<S_{p_{2},p^{*}}$, so the assumption is not valid, $p^{\prime }=p^{*}$ must hold. □

**Figure 5:**
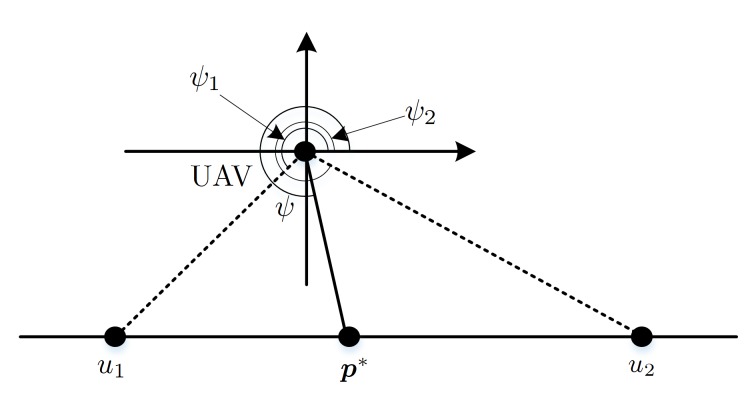
Bounded optimal heading in end-to-end communication.

According to Proposition 1, two conclusions could be given: (1) φ is bounded with $\left(\varphi_{1},\varphi_{2}\right)$, where $\varphi_{1}$ and $\varphi_{2}$ are the horizontal line-of-sight direction of the agents, respectively, and (2) there exists no position out of segment $\left[p_{1},p_{2}\right]$ that is local extreme. Then the gradient direction dir(·) can be bounded with $\left(\varphi_{1},\varphi_{2}\right)$ as shown in Figure 5.

Using the aforementioned aspects, this work propose a bounded gradient-based mobility controller for the UAV in end-to-end communication scenario, where the optimal direction $\varphi_{k}$ can be given as follows:

(39) $$\varphi_{k}=\arg\max_{\varphi_{k}\in \left(\varphi_{1},\varphi_{2}\right)}\frac{\partial J}{\partial p}$$

where

(40) $$\frac{\partial J}{\partial p}=\frac{\partial J}{\partial x}\cos\left(\varphi_{k}\right)+\frac{\partial J}{\partial x}\sin\left(\varphi_{k}\right)$$

Then the UAV flies to the target position using the LGVF guidance law. The pseudocode of the bounded gradient-based mobility control method is given as Algorithm 1.
**Algorithm 1** Mobility Control method for end-to-end communication**Require:**$p_{t_{0}}^{*}=p_{0}$, $\psi_{t_{0}}$. 1: **for**
$t_{k}=t_{1},\ldots ,t_{K}$
**do** 2:   Predict user positions $P=\{p_{i}\}$ at $t_{k+1}$ using Kalman Filter. 3:   Estimate uplink channel parameters with the approximately model defined in Equation (33) using the LSE algorithm, based on observations during time $\left[t_{k-1},t_{k}\right)$. 4:   $\varphi_{k}=\arg\max_{\varphi_{k}\in \left(\varphi_{1},\varphi_{2}\right)}\frac{\partial J}{\partial p}$. 5:   UAV flies towards ${\hat{p}}_{k+1}^{*}$ during time interval $\left[t_{k},t_{k+1}\right]$ with $\dot{\psi }=-K_{l}\langle \eta -\eta^{d}\rangle +\frac{{\dot{\eta }}^{d}}{k_{\eta }\left(\psi \right)}$. 6: **end**
**for**

**Theorem** **2.** *If $v_{\max}>\max\left(v_{1},v_{2}\right)$, the position of the UAV converges to optimal relay position, where $v_{\max}$ is maximum velocity of the UAV, $v_{1}$, $v_{2}$ are the velocities of $u_{1}$ and $u_{2}$.*

**Proof** **of** **Theorem** **2.** Let $v^{*}$ denote the speed vector of $p^{*}$, if the relay UAV desires to converge to $p^{*}$, condition $v>∥v^{*}∥$ should be ensured. If $p^{*}=p_{1}$ or $p^{*}=p_{2}$, then $v^{*}=v_{1}$ or $v^{*}=v_{2}$, respectively. Thus, $∥v^{*}∥=v_{1}$ or $∥v^{*}∥=v_{2}$, which can be satisfied with condition $v_{\max}>\max\left(v_{1},v_{2}\right)$. If $p^{*}\neq p_{1}$ and $p^{*}\neq p_{2}$, then $p^{*}=p_{1}+\lambda \left(p_{2}-p_{1}\right)$ as proved in proposition 1, resulting in $v^{*}=v_{1}+\lambda \left(v_{2}-v_{1}\right)$, where 0<λ<1. Apparently $v^{*}=∥v^{*}∥<\max\left(∥v_{1}∥,∥v_{2}∥\right)$, if $v>\max(v_{1},v_{2})$, then $v>v^{*}$ must hold. □

Theorem 2 shows that the velocity of the UAV is preferred to be faster than the maximum possible velocities of the agents in end-to-end communication situations so as to ensure relay task stability, especially in situations with high-speed ground agents.

### 4.3. Multi-Agent-Inter Communication Scenario

With the estimated channel parameters as described in Section 4.1, and the objective function in Equation (6) is continuous and differentiable, then the optimal relay position in each decision step can be generated by using the gradient method as follows:

(41) $${\hat{p}}_{k+1}^{*}=p_{k}+G*\mathrm{dir}\left(\nabla J\right)$$

where $J=\sum_{i=1}^{N}\frac{1}{S_{p_{i},p}}$. The pseudocode of the gradient-based mobility control method for multi-agent-inter communication shows as Algorithm 2.
**Algorithm 2** Mobility Control method for multi-agent-inter communication**Require:**$p_{t_{0}}^{*}=p_{0}$, $\psi_{t_{0}}$.1: **for**
$t_{k}=t_{1},\ldots ,t_{K}$
**do**2:   Predict user positions $P=\{p_{i}\}$ at $t_{k+1}$ using Kalman Filter.3:   Estimate uplink channel parameters with the approximately model defined in Equation (33) using the LSE algorithm, based on observations during time $\left[t_{k-1},t_{k}\right)$.4:   ${\hat{p}}_{k+1}^{*}=p_{k}+G*\mathrm{dir}\left(\nabla J\right)$.5:   UAV flies towards ${\hat{p}}_{k+1}^{*}$ during time interval $\left[t_{k},t_{k+1}\right]$ with $\dot{\psi }=-K_{l}\langle \eta -\eta^{d}\rangle +\frac{{\dot{\eta }}^{d}}{k_{\eta }\left(\psi \right)}$.6: **end**
**for**

## 5. Simulation Results and Analysis

This section provides simulations to validate the effectiveness of the former proposed mobility control methods, where a smooth turn mobility model [43] is applied to denote the motion of ground users, and the mission environment is classified into four typical types, namely sub-urban, urban, dense-urban and high-rise-urban environments, whose coverage area given in Table 1 and their related channel parameters based on ACM are given in Table 1. Hereby, the channel parameters in Table 2 are experienced data, which can be synthetically referred to in References [29,40,42]. In addition, the fight altitude of the relay UAV is set 400 m in the rest of this section.

Simulations using UAV relay for end-to-end communication and multi-agent-inter communication are executed in the left sections. Synchronously, these simulations test the superiority of the proposed methods by comparing them to other typical methods, namely simplified distance channel model (DCM)-based mobility control method, PCM-based mobility control method.

### 5.1. Static Agents

Using UAV as relays for static agents is a most common situation, where the positions and channel parameters of the agents are assumed prior known to the relay UAV.

In the first simulation, using the UAV as relay for supporting end-to-end communication of two static agents is studied, where the positions of the agents are (370, 2348) and (8701, 2194) in meters, whose transmitter power are 100 mW and 200 mW, respectively, and κ=2.2, $f_{c}=2\mathrm{GHz}$. The initial position of the relay UAV is (3210, 6626) with desired loiter radius 200 m, and $\phi_{\max}=40^{\circ }$. The simulation time is 300 s.

**Figure 6:**
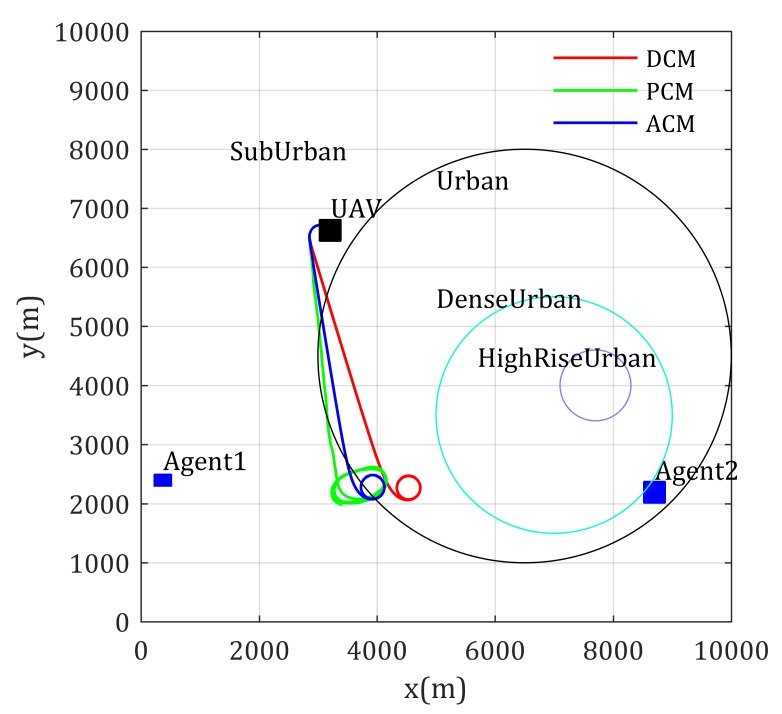
Paths for two static agents end-to-end communication with known channel parameters.

The paths using different channel models are given in Figure 6. The left blue square circle is Agent 1 and the right blue square circle is Agent 2. The circles show the coverage area of different environment type, where the area type inside the purple circle is high-rise urban, the area type inside the cyan circle is dense urban, the area type inside the black circle is urban, the area type of rest environment is sub-urban, and the same settings are used in the rest of this paper.

**Figure 7:**
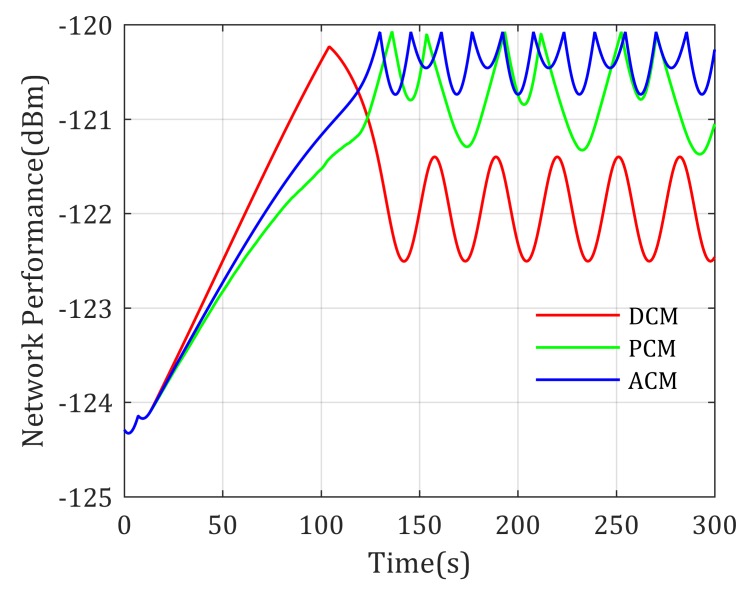
Network performance for two static agents end-to-end communication with known channel parameters.

Simulation results show that the loiter center of these paths are different, where DCM-based mobility control method drives the UAV loiter around the middle point of the two distinguished agents, the PCM-based mobility control method prefers to drive the UAV closer to Agent 1 than DCM-based method because the transmit power of Agent 1 is much smaller than Agent 2. However, according to the experienced channel parameters shown in Table 2 and the ground-to-air channel model in Equation (30), the signal power decreases much faster in urban environments than sub-urban environments, to achieve equal RSS from Agent 1 and Agent 2, the realistic optimal relay position should be closer to urban environments. As shown in Figure 6, the loitering center of the proposed ACM-based mobility control method is closer to Agent 2, which matches the above theoretic analysis. It validates that considering the effects of environment to channel properties is meaningful. Actually, the achieved network performance using these mobility control methods is shown in Figure 7, where the ACM-based mobility method provides the best performance (highest objective value) compared with the other two methods, also reflecting that the ACM is better used in realistic applications.

Then, using the UAV as relay for supporting the inter communication of multiple static agents are simulated, where the channel parameters are prior known to the UAV. The positions of the agents are (2000, 2000), (3500, 8000), (4000, 5000), (8000, 4000), and (6000, 3000). The transmitter power of the agents is randomly given between 100mW and 200mW. The other parameters are set the same as the former simulation.

**Figure 8:**
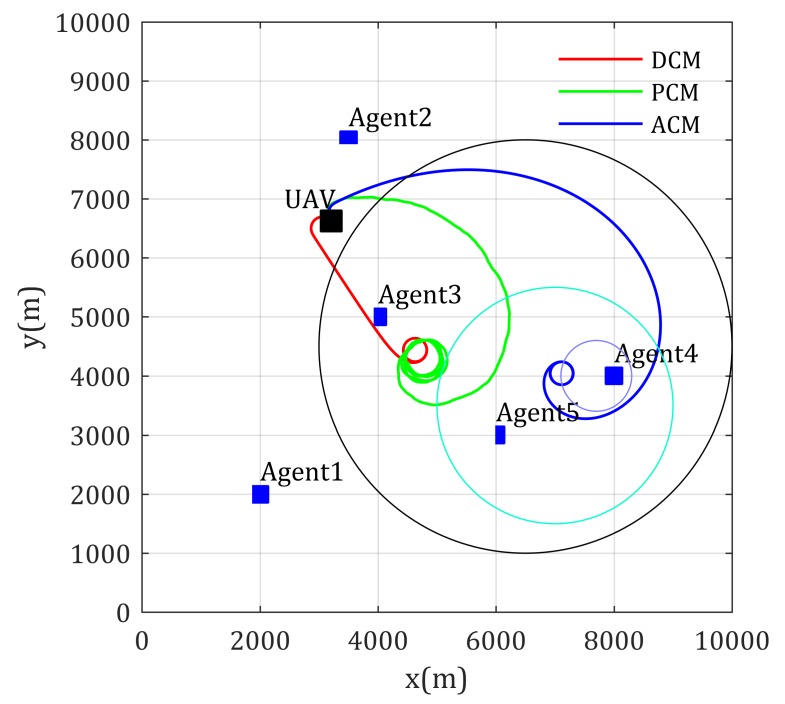
Paths for multi-static-agents-inter communication with known channel parameters.

**Figure 9:**
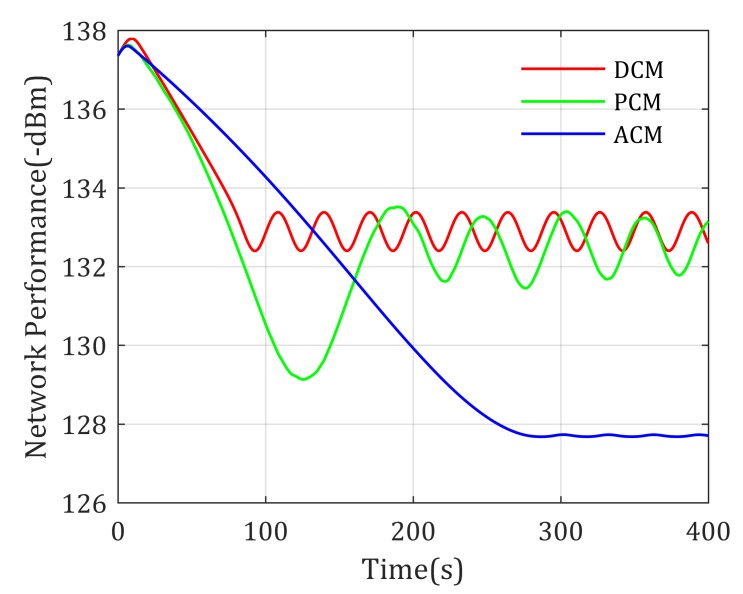
Network performance for multi-static-agents-inter communication with known channel parameters.

Simulation results of the fly paths of the UAV using different mobility control method, and the network performance changing curve are given as Figure 8 and Figure 9, respectively. As Figure 8 shows, Agent 4 is located in a high-rise urban environment, where the communication environment is comparatively worse, because high and dense buildings make LoS components of the channel with low possibility. The blue path shows that the ACM-based mobility control method drives the relay UAV to positions much suitable than the DCM- and PCM-based mobility control methods. The network performance shown in Figure 9 also validates this (lower is better in this figure). Though the path using the ACM-based method is not the shortest path from the UAV’s current position to optimal relay position, its convergence are still guaranteed.

### 5.2. Mobile Agents with Known Channel Parameter

In this section, simulations on mobility control of relay UAV for optimizing the communication of mobile agents are further executed, where the channel parameters are also prior known to the UAV. In the next section, the most realistic situation, where the agents are mobile with unknown channel parameters will be further studied.

**Figure 10:**
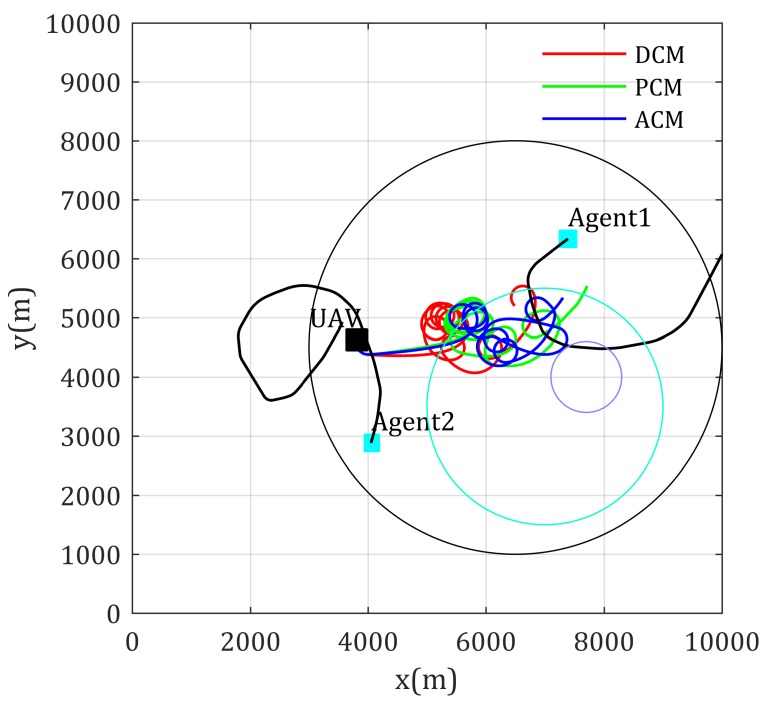
Paths for two mobile agents end-to-end communication with known channel parameters.

**Figure 11:**
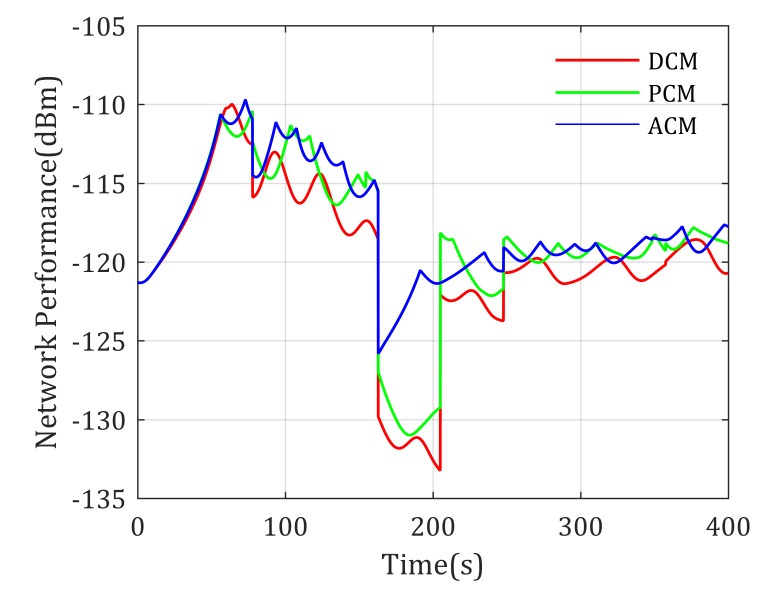
Network performance for two mobile agents end-to-end communication with known channel parameters.

The first simulation considers using UAV as relay for optimizing the end-to-end communication of two mobile agents, whose moving paths are randomly given using the aforementioned smooth turn mobility model. These paths are shown as a cyan line in Figure 10. The transmitter power of the agents is 100 mW and 200 mW, respectively. The initial position of the UAV is (3810, 4626), the velocity is 40 m/s, the maximum bank angle is 40 degree, and desired loiter radius is 200 m. Simulation results of the paths using different mobility control methods and the network performance changing curve are shown in Figure 10 and Figure 11, respectively.

From Figure 11, we can find out that there exists a crash of the network performance; this is because Agent 1 moves into the high-rise urban area, and the channel performance becomes rather bad. The curves show that the ACM based mobility control method gives the best management to the suddenness. In addition, in other times, the ACM mobility control method can also serves the communication of two mobile agents well.

**Figure 12:**
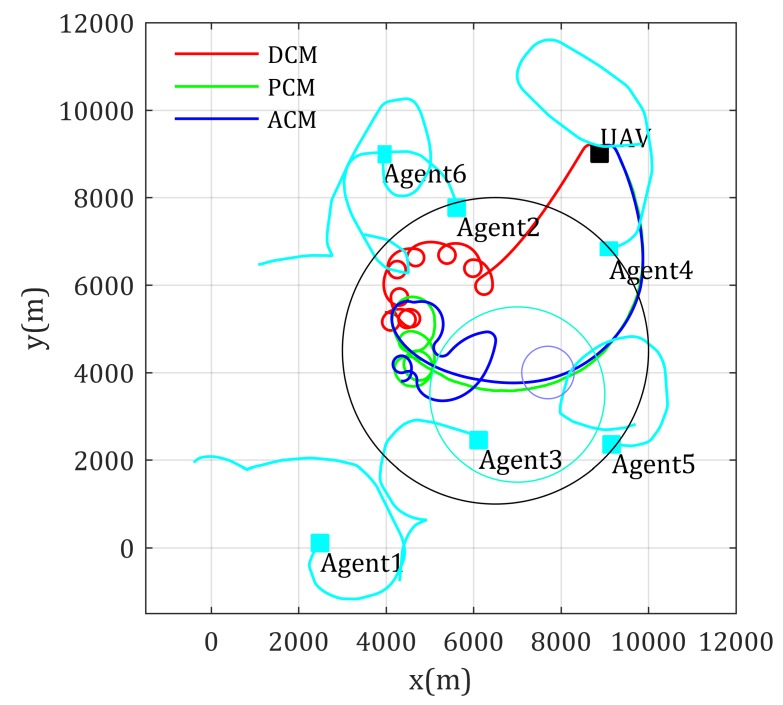
Paths for multi-mobile-agents-inter communication with known channel parameters.

**Figure 13:**
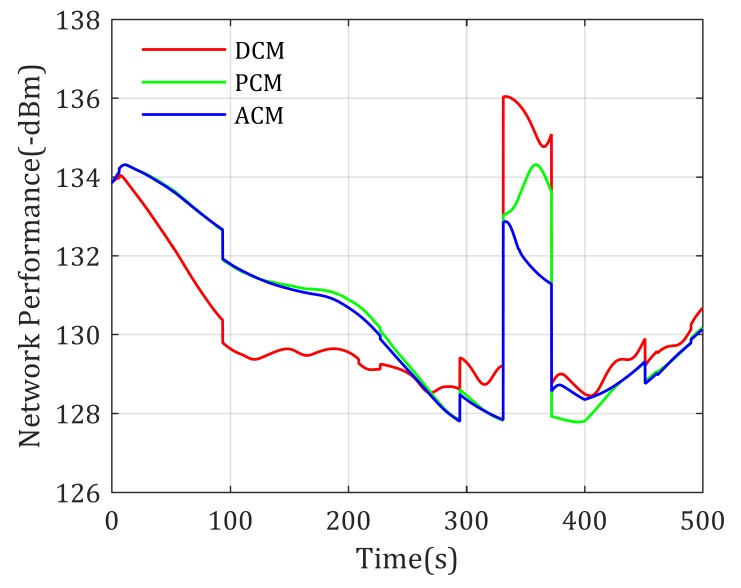
Network performance for multi-mobile-agents-inter communication with known channel parameters.

Similar results in using UAV as relay for multi-agent-inter communication situation are shown in Figure 12 and Figure 13. Inversely, the lower value in Figure 13 gives better network performance. In this simulation, the network performance crash occurs because Agent 5 moves into the high-rise urban area, the RSS from Agent 5 crashes, this in turn makes the network performance objective function, as shown in Equation (6), crashes. Similar to the former simulation, the ACM-based mobility control method gives the best management to such case where agents moving into area with rather low RSS quality.

These simulations show that the proposed mobility control methods are effective to mobile agents relay communication, and synchronously considering the effects of environments to channel properties when controlling the mobility of the relay UAV would give out better network performance, and shows its priority in dealing with sudden cases. Next, simulations on using UAV as relay for mobile agents with unknown channel parameters will be studied.

### 5.3. Mobile Agents with Unknown Channel Parameters

**Figure 14:**
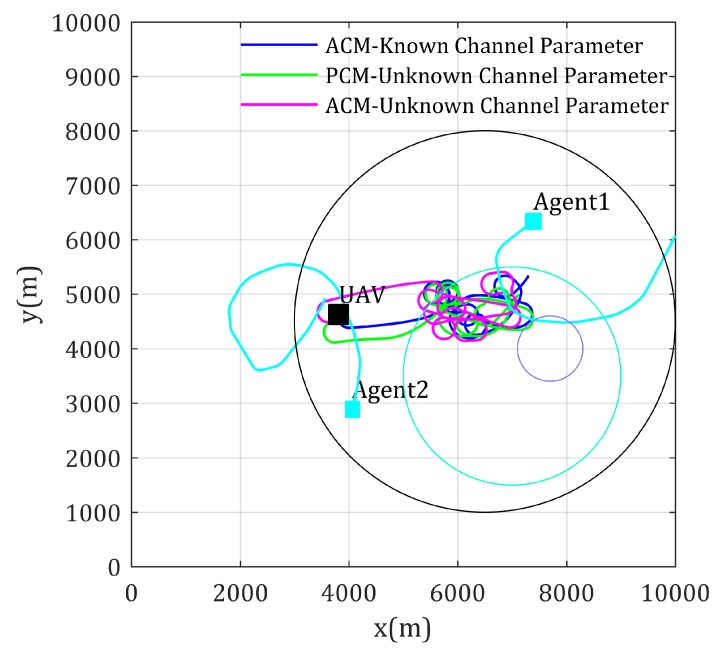
Paths for two mobile agents end-to-end communication with unknown channel parameters.

**Figure 15:**
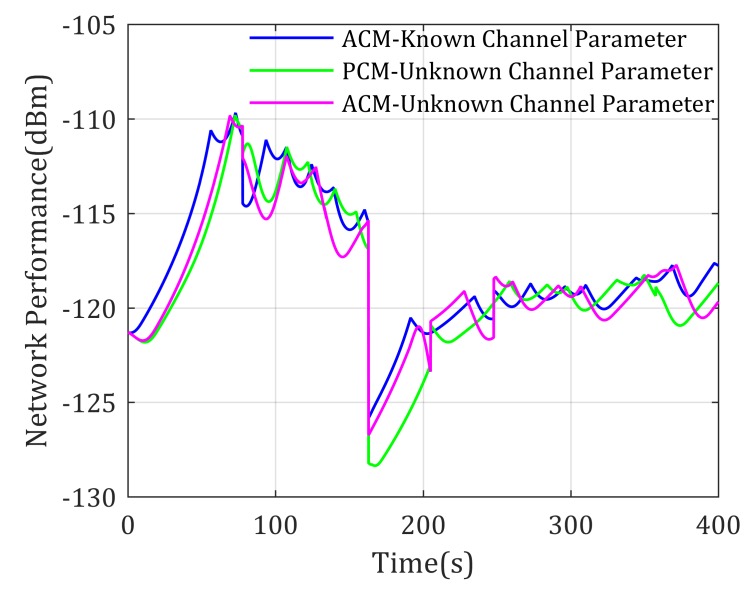
Network performance for two mobile agents end-to-end communication with unknown channel parameters.

This section further considers that the channel parameters are previously unknown to the UAV, which matches the most realistic application, and requires that the UAV estimates the channel parameters and predicts the signal distributions according to limited samples. Let the mobility control methods using ACM with known channel parameters act as the theoretical optimal comparison standard. The performance of the proposed methods based on ACM and approximated channel estimation algorithm (denoted as ACE-ACM here and after) described in Section 5.1 is tested, whose performance is also compared to the mobility control method based on PCM and channel estimation algorithm proposed in Reference [13] (denoted as CE-PCM here and after). Simulation parameters are set the same as in Section 5.2, the only difference is when making decisions, the UAV has no knowledge of the channel parameters of each agents. Figure 14 shows the paths generated by the three mobility control methods, whose performances are shown in Figure 15.

It can be shown that: (1) network performance generated by ACE-ACM and CE-PCM-based methods is worse than the theoretical optimal result, but they are already pretty close and show no fundamental difference; (2) our proposed approximated channel estimation algorithm is effective at estimating ACM based ground-to-air uplinks; (3) ACE-ACM-based methods generate better network performance than CE-PCM, especially when suddenness occurs in the communication network.

**Figure 16:**
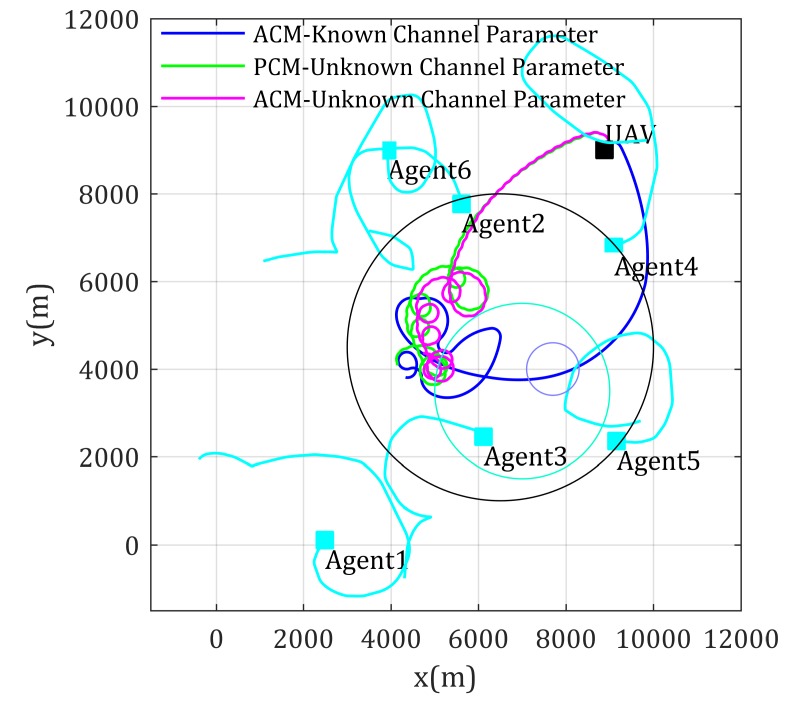
Paths for multi-mobile-agents-inter communication with unknown channel parameters.

**Figure 17:**
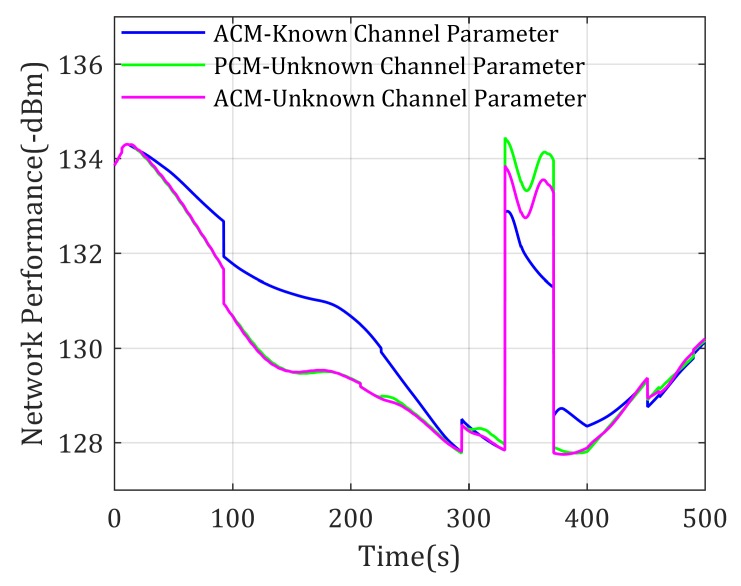
Network performance for multi-mobile-agents-inter communication with unknown channel parameters.

A similar simulation is also carried on using UAV as relay for multi-mobile-agent-inter communication, whose results are shown in Figure 16 and Figure 17. By comparison these figures, similar conclusions and analysis could be obtained as the former simulation.

In summary, the network performance in the above simulation for end-to-end communication with unknown channel parameters using the proposed Algorithm 1 is higher than PCM-based methods and near theoretical optimal when agents enter the environment with poor channel properties. Similarly, the network performance for multi-agent-inter communication with unknown channel parameters using the proposed Algorithm 2 is lower than PCM-based methods and near theoretical optimal when agents enter environment with poor channel properties. In addition, the network performances are eventually closer to theoretical value. These show that the achieved communication qualities using proposed methods are optimal or near optimal, and in turn validate that it is meaningful to consider environment effects when designing the relay UAV mobility control method, as well as validate that the proposed mobility control methods are effective and stable.

## 6. Conclusions

This work studies the mobility control problem of UAV as a communication relay in multi-agent on-demand missions, with considerations of mobile agents and completely unknown wireless channel properties, only using online measured information of received signal strength (RSS) and agent positions. To solve this difficult problem, we concentrate on a framework of model-based mobility control methods to drive the relay UAV to the optimal relay location is proposed, where an LSE-based method is used to estimate channel parameters and gradient-based methods to generate optimal relay position for end-to-end and multi-agent-inter communication, respectively. Simulation results show that the proposed channel approximation algorithm and the mobility control algorithms are effective on driving the relay UAV to arrive at or follow the optimal relay position, so that the optimal network performance is guaranteed. It also reflects that the environment (such as buildings, trees, terrains) affects the channel properties; using mobility control methods while considering these effects generates better network performance.

Future works potentially rely on: (1) extending the single UAV relay to multiple UAV relay situation; (2) studying the mobility control algorithms with collision free formation; (3) extending the proposed mobility framework to 3D area with more sophisticated flight control system model, where the rising, diving, hovering operations, and air affections may be considered, and studying the affections of UAV altitude on communication quality; (4) MIMO and beam-forming technology are beneficial for improving communication quality, where the co-related channel approximation methods are worth studying; (5) the objective functions in this paper are built based on RSS, which is low-level index in wireless communication, mobility control methods for higher-lever communication quality metrics, such as throughput, delay, and QoS, are also worth studying.

## Figures and Tables

**Table 1:** Covering areas of the environments.

Environment	Coverage Center		Coverage Radius (m)
	X (m)	Y (m)	
Sub-Urban	5000	5000	10,000
Urban	6500	4500	3500
Dense-Urban	7000	3500	2000
High-Rise-Urban	7700	4000	600

**Table 2:** Channel Parameters in different urban environment

Channel Parameters	Sub-Urban	Urban	Dense-Urban	High-Rise-Urban
α	0.1	0.3	0.5	0.5
β	750	500	300	300
γ	8	5	20	50
*C*	4.88	9.61	12.08	27.23
*B*	0.43	0.16	0.11	0.08
$\eta_{1}\left(\mathrm{dB}\right)$	0.1	1.0	1.6	2.3
$\eta_{2}\left(\mathrm{dB}\right)$	21	20	23	34
